# Multiomic Approaches to Uncover the Complexities of Dystrophin-Associated Cardiomyopathy

**DOI:** 10.3390/ijms22168954

**Published:** 2021-08-19

**Authors:** Aoife Gowran, Maura Brioschi, Davide Rovina, Mattia Chiesa, Luca Piacentini, Sara Mallia, Cristina Banfi, Giulio Pompilio, Rosaria Santoro

**Affiliations:** 1 Unit of Vascular Biology and Regenerative Medicine, Centro Cardiologico Monzino-IRCCS, 20138 Milan, Italy; davide.rovina@cardiologicomonzino.it (D.R.); Sara.Mallia@cardiologicomonzino.it (S.M.); rosaria.santoro@cardiologicomonzino.it (R.S.); 2 Unit of Cardiovascular Proteomics, Centro Cardiologico Monzino-IRCCS, 20138 Milan, Italy; maura.brioschi@cardiologicomonzino.it; 3 Bioinformatics and Artificial Intelligence Facility, Centro Cardiologico Monzino-IRCCS, 20138 Milan, Italy; mattia.chiesa@cardiologicomonzino.it; 4 Department of Electronics, Information and Biomedical Engineering, Politecnico di Milano, 20133 Milan, Italy; 5 Department of Cardiac Surgery, Centro Cardiologico Monzino-IRCCS, 20138 Milan, Italy; 6 Department of Biomedical, Surgical and Dental Sciences, University of Milan, 20122 Milan, Italy

**Keywords:** muscular dystrophy, dystrophinopathies, dystrophin-associated cardiomyopathy, multiomic analysis, preclinical precision models

## Abstract

Despite major progress in treating skeletal muscle disease associated with dystrophinopathies, cardiomyopathy is emerging as a major cause of death in people carrying dystrophin gene mutations that remain without a targeted cure even with new treatment directions and advances in modelling abilities. The reasons for the stunted progress in ameliorating dystrophin-associated cardiomyopathy (DAC) can be explained by the difficulties in detecting pathophysiological mechanisms which can also be efficiently targeted within the heart in the widest patient population. New perspectives are clearly required to effectively address the unanswered questions concerning the identification of authentic and effectual readouts of DAC occurrence and severity. A potential way forward to achieve further therapy breakthroughs lies in combining multiomic analysis with advanced preclinical precision models. This review presents the fundamental discoveries made using relevant models of DAC and how omics approaches have been incorporated to date.

## 1. Dystrophin-Associated Cardiomyopathy (DAC)

Muscular dystrophy (MD) is an all-encompassing term used to describe rare genetic disorders affecting skeletal and cardiac muscle function frequently caused by mutations in the dystrophin gene (*DMD*) [1]. Inherited or spontaneous *DMD* gene mutations result in absent or defective truncated dystrophin protein expression. Dystrophin is a high molecular weight protein (427 kDa) encoded by a large gene (79 exons), it is localised under the sarcolemma and it is connected to the multi-protein complex called the dystrophin-glycoprotein complex (DGC) which together links the intracellular actin cytoskeleton with the extracellular matrix (ECM). These multi-protein interactions underlie the integrative dual function of dystrophin as a mechanical stabiliser and a signalling platform that is essential for orchestrating ion channel activation, reactive oxygen/nitrogen species (ROS/RNS) release, membrane lipid arrangement, G-protein-coupled receptor and other sarcolemma-associated protein assemblies [2]. Consequently, dystrophin deficiency deregulates cell-signalling and makes myofibres sensitive to mechanical damage, leading to myocyte death and loss of tissue function. In particular, in cardiac tissue the dystrophin deficiency is associated with myocarditis, fibro-fatty substitution, cardiomyocyte death, left ventricular dysfunction, arrhythmias, dilated cardiomyopathy and heart failure (HF) [3,4].

There is considerable heterogeneity in clinical presentation of dystrophinopathies. For example, in the most common early onset severe form, named Duchenne muscular dystrophy (DMD) [5], out-of-frame *DMD* mutations disrupt full mRNA translation, thus no dystrophin can be expressed. In contrast, in-frame mutations, such as Becker muscular dystrophy (BMD), do not fully block dystrophin production but rather result in the expression of truncated dystrophin isoforms of varying size and functionality. However, the reading frame rule often does not explain the patients’ phenotype, especially in the cases with cardiomyopathy involvement [5]. In the pre-steroid era DMD and BMD had a predictable disease projection with premature death in the early twenties or forties respectively. However, opportune treatment advances, e.g., nocturnal ventilation and corticosteroid regimens, opened a new era of treatment [6,7] which necessitates considerable planning particularly regarding cardiac care but also the choice of outcome measures for clinical studies or preclinical models.

**Figure 1:**
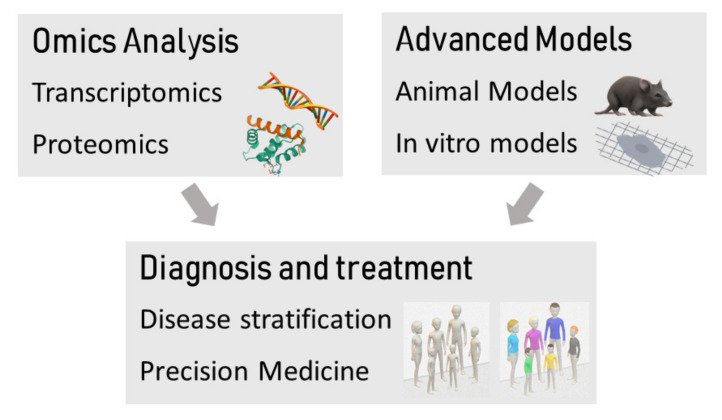
Combining omics technologies with advanced models can support a precision medicine approach, leading to the development of novel treatments.

Dystrophin-associated cardiomyopathy (DAC) phenotypes are clinically heterogeneous e.g., age of onset, progression rates and severity [8,9,10]. Numerous studies have failed to determine distinct genotype-phenotype correlations or an underlying mechanism to explain these heterogeneous dystrophic cardiac phenotypes [11]. Furthermore, although gene-targeting approaches (e.g., restoring partial dystrophin expression) and pharmacological treatments (e.g., corticosteroids) reduce skeletal muscle disease and prolong ambulation, they do not provide clear benefits for the heart. Thus, new therapeutic strategies need to be undertaken in order to improve the prognosis of patients with MD and cardiac involvement. Overall, the causal mechanisms defining the final cardiac phenotype likely involve nuanced activation of known and novel complex pathogenic mechanisms acting collectively [11]. This will, without doubt, require more precise targeting and increased modelling resolution (Figure 1). Great encouragement has come from omics studies which to date have provided significant knowledge on the spectrum of *DMD* mutations, the involvement of modifying genes, the alteration of pathogenic signalling mechanisms and the identification of biomarkers that have supported diagnosis and monitoring the impact of therapeutic care.

## 2. Omics Application in DAC Searching for Insight

The progress in high-throughput technology (HTT), such as proteomics, genomics and metabolomics has provided new opportunities to understand the pathophysiology of complex diseases and generate large amounts of data stratifying different molecular levels. Proteomic HTT analysis can currently examine proteins in detail (e.g., membrane, cytosol, microsomal, or nuclear fractions) down to the femtomolar level producing information on protein post-translational modification, cellular location and function. Transcriptome profiling, which enables a large-scale investigation of pathological processes in diseased tissues, is a well-established and effective method of dissecting and analysing specific phenotypes. Any changes occurring in response to different healthy or diseased states, specific genetic and epigenetic programs, or environmental challenges can leave a typical signature that is detectable at the transcriptome level. Metabolomic profiling which aims to measure the end products of all of the biological systems’ processes in a ‘here and now’ context, can provide real-time functional data. To appreciate the complexity of systems biology, the omics disciplines adopt a holistic view rather than a hypothesis-driven approach. In other words, omics exploit the simultaneous measurement of a huge number of features (e.g., genes, proteins or metabolites), without an a priori selection of the factors of interest which distinguishes omics from the reductionist approach focused on analysing one or a few known, pre-defined variables. The unbiased analysis of a bulk of variables through omics techniques allows the development of hypotheses that can be subsequently tested and validated.

The opportunities enabled by omics analyses concern a broad spectrum of applications e.g., studying the pathophysiology of a specific disease along with potentially revealing unrecognised causes of disease onset or progression, reconstructing gene/protein-regulatory networks, characterizing gene expression dynamics, identifying putative disease-sensitive targets, finding disease biomarkers for diagnosis, stratifying patients’ prognosis and ultimately translating such knowledge into clinical applications. Integration of these data sets into multiomics data means that thousands of genes (genomics), RNAs (transcriptomics), proteins (proteomics) and metabolites (metabolomics) can be studied simultaneously, also revealing their interaction networks.

Thus, a comprehensive omics approach has the potential to provide useful insight into DAC pathogenesis and produce a detailed molecular picture enabling the stratification of patients into well-defined groups in terms of choosing the best disease-management strategies and treatments, which is the basis of precision medicine [12]. Lastly, omics might also provide early diagnostic or prognostic information to improve patient care and prevent adverse outcomes. Thus, identification of biomarkers sensitive and specific enough to measure clinical benefit would enable a smoother path to demonstrate potential efficacy for particular interventions. This eventuality could improve the journey of translational research along the ‘bench-to-bedside’ pipeline to speed up bringing effectual and safe therapeutics to suitable patients.

## 3. Genome-Editing-Based Models of DAC

Currently, it is estimated that between 30 and 60 models involving *DMD* mutations have been developed from non-mammalian (e.g., *C. elegans, D. melanogaster* and *D. rerio*) and mammalian models (e.g., *M. musculus, R. norvegicus, O. cuniculus, F. catus, C. familiaris* and *S. domesticus*) [13,14,15]. In this section we focus on models that were specifically genetically edited with the aim to better recapitulate DAC. Indeed, several of these models have been investigated via omics analyses, however these studies have mainly focused on skeletal muscle disease rather than cardiomyopathy.

Genome-editing technology has proven to be the most promising way to enhance both animal and patient-specific preclinical DAC models to unprecedented levels of affinity to humans and compatibility with HTT analyses for disease modelling and drug screening. Such intentionally modified models are fundamental to understanding the influence that genetic variance has on genotype-phenotype correlations and to evaluate the effectiveness of gene-modifying therapies. Using gene editing it is theoretically possible to generate tailored models containing a specific desired mutation in the *DMD* gene or in additional genes, thus enabling the possibility to describe correlations between genetic background and disease phenotype.

In particular, the discovery of the clustered regularly interspaced short palindromic repeats (CRISPR) and CRISPR-associated proteins (CRISPR/Cas) was a millstone for the development, application and accessibility of site-specific gene editing. Unlike zinc-finger nucleases (ZFNs) and transcription activator-like effector nucleases (TALENs), CRISPR/Cas creates site-specific DNA double-strand breaks using short guide RNA sequences, making the design and use of this tool easier with respect to ZFNs and TALENS.

Despite being the canonical DMD model, *mdx* mice do not fully recapitulate the human phenotype since they do not develop cardiomyopathy and only have a 25% reduction in lifespan [16,17]. Gene editing has made it possible to recapitulate human *DMD* mutations in murine models that are more representative of the clinical scenario, which in turn can also be used to evaluate the therapeutic value of gene targeting drugs. The majority of these murine models were generated via CRISPR/Cas9 editing to induce exon deletions [18,19,20,21,22] or insertion/deletion (indel) mutations [23]. Other editing approaches, e.g., base editing and TALENs, have also produced point mutations [24] and exon deletions [25] in *mdx* mice. A little over half of the reported models (5/8 reports) assessed cardiac tissue which determined the lack of cardiac dystrophin expression [18,19,20,22] and variation in myofibre size, signs of inflammation and presence of infiltrating immune cells [21]. These disease readouts were sufficient to include a therapeutic research arm, i.e., screening the efficiency of CRISPR/Cas9-mediated reframing or antisense oligo nucleotide (AON)-targeted exon skipping in all but one report [21]. Similar CRISPR/Cas9 approaches have been used to generate rat [26,27,28,29], rabbit [30] and non-human primate [31,32] models of dystrophinopathy that showed progressive heart involvement similar to those observed in humans.

As earlier briefly mentioned, genome-editing tools have been also applied to correct dystrophin mutations in different animal models including mouse, rabbit, dog and pig. The development of fully ‘curative’ genetic therapies for patients with *DMD* mutations is very challenging due to the large size of the *DMD* gene (2.2 Mb) and its transcript mRNA (14 kb) that cannot be inserted into any known vector. Additionally, the approach of gene editing to completely revert *DMD* mutations is also complicated since the majority of patients have deletions of one or more exon [33]. However, different genome-editing approaches aimed at restoring dystrophin expression were developed and have already been used in both in vitro and in vivo models [33].

Several studies have demonstrated the ability of CRISPR/Cas9 technology to delete out-of-frame *DMD* exons, restoring the expression of semi-functional dystrophin protein in vivo [19,34,35,36,37,38]. In other works, homology-directed repair was used to restore low-level dystrophin expression in *mdx* and other murine dystrophinopathy models [37,39,40,41,42]. CRISPR/Cas9 editing approaches were also used to correct *DMD* exon mutations of increasing complexity animal models, including mice [24], dogs [43], pigs [44], etc. All these experiments support the idea that, these models are suitable for screening novel *DMD* gene-targeting therapies and, following further development for improved safety, gene-editing tools, in particular, may be applied to treat dystrophinopathy patients and ameliorate their quality of life.

Examples regarding the high relevance of the application of omics approaches to the dystrophinopathy models mentioned above are described in the following paragraphs.

## 4. Proteomic Investigations of Dystrophin Deficiency

Several proteomic preclinical studies performed in the past decades unveiled some of the molecular pathophysiology of dystrophinopathies (Table 1). The first studies were mainly based on protein separation by 2D gel electrophoresis coupled to mass spectrometry (MS) for identification of regulators [45,46,47]. However, advancements in the MS field paved the way for the application of gel-free approaches characterised by highly accurate peptide identification and quantitation [48]. Carr et al. identified 31 proteins that are commonly modulated in different dystrophin-deficient tissues by integrating data obtained from nine MS-based studies focused on tissue-specific proteomes of different murine, canine and porcine models of DMD. The proteins identified were those mainly involved in maintaining the actin cytoskeleton or in regulating cellular energy metabolism. In particular, alterations in bioenergetics and mitochondrial functions were shown in skeletal muscle, while increased fibrosis was identified in the diaphragm. The same authors also summarised the results of studies on urine and blood samples designed to search for potential circulating diagnostic and prognostic biomarkers that would be more specific than blood creatine kinase and less invasive than muscle biopsies. They identified 33 circulating proteins modulated by DMD, including leakage proteins associated with muscle damage, connective tissue remodelling, or proteins involved in inflammation [48].

Notably, a different label-free MS-based proteomic approach comparing the urinary proteome of MD patients (DMD, BMD and limb-girdle MD) versus healthy subjects, identified titin fragments as the most promising urinary biomarker for MD screening [49]. These results were also confirmed by enzyme-linked immunosorbent assays in *mdx* mice at different ages, in which *N*-terminal titin fragments showed a dramatic increase coincident with muscle damage. Furthermore, urinary levels of titin were higher in DMD than in BMD patients [50]. Thus, urinary titin is now considered as a very promising non-invasive biomarker not only for MD screening, but also for severity prediction [51,52].

Finding circulating biomarkers for identifying therapy-responsive patients is another important need that is particularly relevant due to the increasing number of candidate drugs entering clinical trials and the lack of optimal outcome measures adaptable to the contemporary natural history of MD [6,7]. In this context, two fragments of the myofibrillar structural protein myomesin-3 were identified to be abnormally increased in the serum of MD patients (DMD and limb-girdle MD type 2D) using a comprehensive high-resolution MS-based approach, and they are thus proposed as biomarkers for assessing experimental therapies for MD and other neuromuscular disorders [53]. Furthermore, the potential use of distinct chaperones such as cardiovascular heat shock proteins (HSP), HSPB7, HSPB5 (αBC), HSP70 (HSPA) and HSP90 (HSPC) as dystrophin deficiency disease markers for secondary pathological changes or for the evaluation of novel treatments was also recently proposed [54].

Several proteomic studies have also focused on DAC in animal models of dystrophinopathies with the final aim of identifying biomarkers for improved diagnostic procedures, prognosis of important complications and evaluation of novel drug or cell-based treatments [55]. Initially, gel-based proteomic approaches were applied to study alterations of the myocardium in *mdx* mice. For instance, Gulston et al. compared wild type (WT) and *mdx* mice at different ages (1-, 3-, 5-, 7- and 9-month-old mice) by two-dimensional difference gel electrophoresis (2D-DIGE) in association with nuclear magnetic resonance (NMR) metabolomic analysis [46]. This multiomic data enabled the classification of heart tissue as degenerative at all the time points except for 1-month-old mice. The most pronounced changes observed in 5-month-old mice that were primarily attributed to mitochondrial and glycolytic enzymes [46]. Lewis et al. also applied the 2D-DIGE approach to analyse heart tissue from 9-month-old WT and *mdx* mice, and identified 26 altered proteins involved in energy metabolism, cardiac contraction and cytoskeletal organisation [47]. In particular, a drastic reduction of numerous mitochondrial proteins and metabolic transporters was detected, suggesting that alterations in the mitochondrial proteome might influence other cellular functions, involved in myofibre contraction making the *mdx* heart more susceptible to damage and fibrosis [47,48,49,50,51,52,53,54,55].

Adopting a label-free MS-based proteomic approach, Holland, Dowling et al. compared control and *mdx* mice hearts prior to the occurrence of extensive cardiac damage (i.e., in 7-week-old mice) and when profound cardiac dysfunctions were evident in 20-month-old mice [56,57]. Results from this study clearly demonstrated increased transferrin and immunoglobulin expression, the latter being probably responsible for an autoimmune response to the degenerating myocardium. As expected, a marked reduction of proteins involved in stabilising the basal lamina and organising the cytoskeletal network, fibre contraction and energy metabolism were also identified. Specifically, the most altered proteins were laminin, nidogen and annexin, which increased with age but were dramatically reduced in 20-month-old *mdx* mice. These studies formed the hypothesis that the absence of dystrophin alters the DGC that consequently affects basement membrane components such as reduced nidogen (a sulphated glycoprotein present in basement membranes with a role in cardiac and lung development) and annexin (responsible for Ca^2+^ homeostasis and cytoskeleton and ECM maintenance). Together these alterations could be the trigger for progressive fibrosis that characterises the dystrophic deficient heart [57].

These results were partially confirmed by a study that compared senescent 20-month-old *mdx-4cv* mice with age-matched WT mice, by means of a MS-based proteomic approach [58]. Murphy et al. demonstrated the complete absence of the full-length dystrophin isoform and a significant reduction of the dystrophin ligand α-syntrophin and other protein components of the DGC in the heart. Furthermore, the study also showed distinct alterations in basal lamina components, Ca^2+^-binding proteins (e.g., sarcalumenin), proteins of the ECM (e.g., periostin), proteoglycans (e.g., lumican), cardiac-specific myosin light chain kinase, proteins involved in the response to stress (e.g., HSPs) and a large number of mitochondrial and glycolytic enzymes, confirming previous results in *mdx* mice. Notably, they also demonstrated a decrease of laminin, confirming the hypothesis that this protein could trigger whole-organ alterations [56]. Interestingly, Johnson et al. specifically analysed the DGC in the skeletal muscles and hearts of 20-week-old *mdx* mice by immunoprecipitating the protein complex using an antibody directed against a portion of dystrophin not involved in the interaction with other known members of the complex that were later identified by MS [59]. A total of 15 proteins were detected in skeletal and cardiac muscles that had reduced association with dystrophin, such as neuronal nitric oxide synthase (NOS) and beta-1-syntrophin in the heart. Furthermore, they identified novel cardiac-specific interactors, including cavin-1, crystallin alpha B, anhak1 and cypher, suggesting a direct link between dystrophin and two major cardiac signalling mechanisms that are disrupted in dystrophin deficient hearts i.e., the regulation of ion flux and sarcomeric contraction [59].

Dystrophin deficient muscle pathophysiology also involves increased intracellular nitrosative stress [60] caused by NOS delocalisation following the loss of dystrophin-sarcoglycan linkage and increased NOS activity due to high levels of Ca^2+^ coupled with hyper-activation of the nonselective cation channel transient receptor potential canonical channel 6 (Trpc6). Notably, selective pharmacological suppression of Trpc6 or TRPC6 gene deletion normalises mechanosensitive Ca^2+^ and force responses in young mice. Following on from this, Chung et al. focused attention on nitrosylated proteins in heart tissue of *mdx* versus WT mice with or without the deletion of TRPC6 [61]. The application of a dual labelling MS-based proteomic approach allowed them to selectively enrich more than 1200 nitrosylated peptides, most of which were present in both *mdx* and WT mice, but with a higher abundance in *mdx* mice. The majority of myocardial proteins with increased nitrosylation were mitochondrial or sarcomeric proteins e.g., the important antioxidant defence protein peroxiredoxin. Interestingly, TRPC6 deletion reversed the nitrosylation of 70% of the identified peptides in addition to improving cardiac dysfunction and remodelling. It can be surmised that deleting a pathological source of Ca^2+^ (i.e., through Trpc6 pharmacological inhibition or genetic deletion) can rebalance the NO-ROS axis and reduce S-nitrosylation [61]. Results from this study suggested a deleterious role for increased S-nitrosylation in DAC which contrasts a protective effect previously observed in an ischaemic injury model via the protection of specific cysteine residues from irreversible oxidation [62]. However, it has also been demonstrated that excessive S-nitrosylation can contribute to the pathogenesis of other diseases e.g., Alzheimer’s and Parkinson’s diseases [63].

Since *mdx* mice do not completely recapitulate DMD, particularly DAC, Tamiyakul et al. performed a proteomic study on the porcine DMD model [64]. Using an isobaric tag for relative and absolute quantitation (iTRAQ) quadro plex-based proteomic approach coupled with OFFGEL pre-fractionation of peptides before MS analysis, they compared heart tissue from WT and DMD pigs at different ages (2-day-old representing an early pre-symptomatic stage and 3-month-old to account for a more advanced disease stage). In the myocardium of 3-month-old DMD pigs they found a decreased abundance of several mitochondrial proteins, e.g., those involved in beta-oxidation and respiratory chain functions, indicating impaired mitochondrial energy production. This is in line with previous findings obtained in *mdx* and *mdx-4cv* mice. In parallel, increased levels of acute-phase proteins, which are associated with the inflammatory response, were also detected. In 2-day-old and 3-month-old pigs, they also showed that several proteins playing an important role in heart muscle function were altered and that many ribosomal proteins were markedly reduced, suggesting decreased translation activity connected to significantly reduced heart weight and cardiomyocyte diameter. Furthermore, decreased levels of DGC components indicate sarcolemma destabilisation and impaired signal transduction. A direct comparison of the degree of proteome alterations in skeletal muscle and heart tissue indicated that DAC progression is not only slower compared to skeletal muscle but also characterised by different biological and biochemical alterations [64].

Overall, proteomic studies performed in the past two decades in different animal models highlight that aged hearts of dystrophin-deficient animals display impaired mitochondrial metabolism, altered Ca^2+^-flux, basement membrane deficiencies, ECM changes and high cell stress responses. The absence of dystrophin in cardiac fibres appear responsible for the degeneration of contractile myocytes followed by the replacement with non-contractile cells that cause severe functional decline [65,66]. Lastly, mitochondrial dysfunction in combination with Ca^2+^-dependent activation of proteolytic processes are among the most important mediators of myofibre degeneration and fibrosis initiators in the dystrophin-deficient heart [67].

State-of-the-art proteomic approaches have been implemented for protein biomarker identification and mechanistic insights using animal models of dystrophin deficiency. Their translation to human models, particularly models of DAC, should be considered as a new frontier given the differences observed in skeletal versus cardiac muscle.

## 5. Transcriptomic Approaches to Uncover DAC Pathological Mechanisms

Despite its recognised value for inferring pathogenetic mechanisms underlying cardiovascular diseases [68,69,70], transcriptome profiling has not been substantially employed for studying or characterising the cardiac involvement in various types of dystrophin-deficient models.

Rather, the usefulness of transcriptome profiling in the context of dystrophinopathies was primarily supported by microarray analyses of skeletal muscle pathophysiology [71,72,73,74,75,76,77,78,79]. Both patients’ biopsies and animal models have been used to infer the molecular mechanisms underlying particular dystrophinopathies, offering useful insights into the connection between a specific genetic background and the disease phenotype through the transcriptome footprint.

Chen et al. compared skeletal muscle tissue from control donors and patients with dystrophin or alpha-sarcoglycan deficiencies, providing a description of common and unique variations in gene expression for these two forms of dystrophinopathy [71]. Whereas one aspect mainly concerned the levels of dystrophin expression, the authors identified several shared differentially expressed genes (DEGs) suggestive of diverse pathophysiological aspects of the disease such as, those affecting mitochondrial function, energy metabolism dysregulation, calcium-regulated signalling, muscle fibre developmental program, dedifferentiation of dystrophin deficient muscle and inflammatory signature and immune cell infiltration i.e., dendritic and mast cells. Similarly, Haslett et al. found that DEGs in skeletal muscle biopsies from DMD patients were related to muscle structure and regeneration, immune response and ECM compared to unaffected controls [72].

In order to characterise temporal specific gene expression patterns of the pre-symptomatic phase of DMD and define the temporal molecular signatures of DMD progression, Pescatori et al. worked on skeletal muscle biopsies from very young subjects (<2 years) affected by DMD and compared them to biopsies from children with DMD older than 5 years who represent a more advanced stage of disease [73]. The authors proposed that DMD muscle is programmed early to express a distinct gene expression pattern that differs significantly from that of the age-matched muscle biopsies taken from unaffected children. The molecular signature of the dystrophic phenotype was typified by a co-ordinated activation of genes involved in the inflammatory response, ECM remodelling and muscle regeneration, as well as decreased transcription of genes involved in energy metabolism. By analysing DMD time-course biopsies, the authors further demonstrated that certain genes, including members of three morphogenetic signalling pathways (Wnt, Notch and bone morphogenetic proteins) were gradually induced or repressed during the natural history of DMD. Other studies highlighting pathogenetic mechanisms of DMD have been based on the already mentioned *mdx* mouse model. Molecular characterisation of the *mdx* phenotype, including its intrinsic propensity to regenerate skeletal muscle, or of disease progression itself, appear to mirror those involved in humans, for example those related to immune response and inflammation, cell adhesion, muscle structure and regeneration and ECM remodelling [74,75,76,77,78].

Despite the shared aetiology and these mechanistic similarities, it is important to note that the phenotypic differences between DMD patients and the *mdx* mouse model (e.g., presentation of minimal clinical symptoms and a small reduction in lifespan in *mdx* mice) suggest that disease severity may depend in large part on a different response of various skeletal muscles to dystrophin deficiency (both ‘between’ and ‘within’ humans and mice) [79].

Although the use of microarray platforms is still employed, the technological advancement of next generation sequencing (NGS) has greatly improved the quality of measuring RNAs over probe-hybridisation-based technology. RNA-sequencing (RNA-seq), the NGS technique used to profile RNAs, has a broader dynamic range of RNA quantification and allows simultaneous detection of nucleotide variation at the RNA sequence level (e.g., expressed single nucleotide polymorphisms, insertions or deletions) and allelic-specific expression. This enables the reconstruction and identification of novel transcripts, gene fusions or splice variant isoforms which cannot be directly detected at genome level [80]. In other words, RNA-seq captures a level of complexity of the transcriptome that probe-based microarray cannot.

The increasing widespread use of NGS in the last 15 years has further accelerated the evolution of advanced methods that allow an even more precise and detailed assessment of RNAs even at single-cell and tissue spatial resolution. Compared to traditional bulk RNA analysis, which represents an ‘averaged’ RNA signal from the different cells within the tissue, single-cell RNA (scRNA)- and single-nuclei RNA (snRNA)-seq and spatial transcriptomics have paved the way to study cell-tissue heterogeneity, enabling the RNA profiling of rare cells, the discovery and description of previously unknown cell types or subtypes and the quantification and visualisation of the spatial distribution of RNAs within tissue sections [81,82,83,84].

Van Pelt et al. used a multiomics approach, including RNA-seq transcriptome profiling, to compare TA muscles from male *mdx*/*mTR* mice, which lack both a functioning dystrophin and a telomerase RNA component, with those from WT mice. The authors highlighted a number of pathogenic alterations in protein synthesis and degradation, muscle fibre contractility, cytoskeletal architecture, ECM and skeletal muscle metabolism, with dystrophic muscles exhibiting increased glycolytic metabolites. RNA-seq results also suggested that an inflammatory component, fibrotic expansion of the ECM and matrix metalloproteinase activity are responsible for the pathological increase in muscle mass observed in *mdx/mTR* mice [81].

Using normal and dystrophic tibialis anterior (TA) muscles of mice lacking *DMD* exon 51 (ΔEx51), Chemello et al. [85] assessed by RNA-seq and snRNA-seq the transcriptional abnormalities and heterogeneity associated with individual myofibre nuclei that contribute to DMD skeletal muscle pathology. The authors found large gene expression differences between TA muscles from ΔEx51 and WT mice, with cytokine production, inflammatory response and apoptotic signalling the most prominently altered pathways in ΔEx51 TA muscles, whereas genes down-regulated in ΔEx51 TA muscles were related to growth stimulus and development of healthy skeletal muscle. The use of snRNA-seq was motivated by the fact that the syncytial nature of skeletal muscle poses issues about the degree to which individual nuclei may exhibit transcriptional diversity. By comparing different cell populations in the skeletal muscles of DMD and WT mice by snRNA-seq Chemello et al. were also able to identify heterogeneous nuclei populations that were grouped into three main clusters, namely myonuclei of myofibres, nuclei of the regenerative pathway and nuclei of mononucleated cells of skeletal muscle, each characterised by its own transcriptional heterogeneity and expression of specific myofibre isoforms. This study is one of the first in this field that clearly demonstrates the advantages of using gene expression profiling via RNA-seq and snRNA-seq to explore complex tissues in order to more precisely unravel disease-related phenotypes at single cell/nuclei resolution. This ground-breaking study strongly motivates the use of snRNA-seq-based approaches for studying DAC, which could identify novel evidence to understand the physiological changes causing DAC.

Another way of successfully applying RNA-seq is as a clinical diagnostic tool. Although the great technical advance of NGS has improved genetic diagnostics for patients, e.g., the use of whole-exome sequencing, a critical issue in the dystrophinopathy field is the number of cases that still remain with unidentified mutations despite extensive genetic analysis. In this context, RNA-seq approaches have been proposed as a reliable method for detecting novel mutations, thus improving the diagnosis and indexing of dystrophinopathies. Indeed, Gonorazky et al. identified a deep intronic (non-coding) mutation in the *DMD* gene of a MD patient by RNA-seq, highlighting the potential of RNA-seq to identify mutations occurring outside the coding exonic sequence of the *DMD* gene but also those affecting RNA expression or processing [86] that lead to a pathological phenotype.

Despite its indisputable potential, it must be pointed out that transcriptome profiling, either by bulk RNA-seq or sc/snRNA-seq, in the context of DAC has not yet gained much attention. Nevertheless, there could be plausible reasons for the lack of studies implementing these technologies. One possible explanation is that the most outstanding and innovative platforms for NGS, including those for scRNA-seq and spatial transcriptomics, are still very expensive and require a high technical and analytical level of expertise which limits their extensive use, at least for the present. Another major obstacle related to the study of DAC by transcriptome profiling is certainly the difficulty of obtaining human diseased cells/tissues through non-invasive methods. Optionally, in vitro modelling of DAC could be achieved by differentiating heart-like organoids from primary cells or induced pluripotent stem cells (iPSC) derived from patients to investigate subject-specific characteristics of diseased tissue and test putative therapeutic strategies, by either pharmacological or gene therapies. RNA-seq- and sc/snRNA-seq-based analyses could also serve this setting to both monitor the effects produced by therapeutic interventions and provide novel insights on the affected processes downstream of dystrophin deficiency that contribute to skeletal or cardiac muscle pathology [87]. 

A further possibility to study dystrophinopathies is represented by the use of surrogate tissues accessible through minimally invasive practices and often as part of regular clinical visits, e.g., blood, that could provide important information on the immunological status reflecting a specific disease state. This could be extremely relevant for the comprehension of the immune response in dystrophinopathies and particularly for understanding how it shapes and is related to different phenotype or modifies specific therapeutic interventions, such as assessing immune reactions to exon skipping or gene therapy. Modulating inflammation or controlling pathogenic adaptive immune activity by inducing immunological tolerance to de novo dystrophin expression are, in fact, two options that are assumed to be fundamental for applying successful dystrophin-rescue therapies. In this framework, both whole blood transcriptome profiling and specific T and B immune cell repertoire sequencing could effectively describe the relationship between adaptive immune profiles and DAC phenotypes at a systemic level, reflecting the local immune responses and could offer novel opportunities for clinical evaluation and intervention [88,89,90].

The genomic analyses, particularly transcriptomics carried out to date (Table 2), have the capability to unveil distinct genotype-phenotype relationships and characterise underlying DAC mechanisms that explain the heterogeneous cardiac phenotypes. New studies based on genome-wide approaches aimed at unravelling these genotype-phenotype associations are needed to improve our understanding of the mechanisms that ultimately determine DAC therefore enhancing patient care.

## 6. Bioinformatics for Omic Data Integration

Technological progress in the field of biotechnology has undoubtedly contributed to the development of increasingly sophisticated HTT platforms that are continuously generating new data. While progress in understanding biological systems is made, the complexity, volume and resolution of the generated data can also challenge scientists’ ability to efficiently manage the amount of data. Tackling this challenge requires continuous refinements in analysis tools, software design and pipeline development that provide adequate means to answer biological and clinically relevant questions.

Computational approaches for analysing high-dimensional data have advanced considerably since the aforementioned foundational studies and a broad variety of elaborative bioinformatic resources are now available as open source tools enabling any researcher to infer physio-pathological mechanisms from multiomic data. One of the most important resources is the ‘Bioconductor’ project that provides core data structures and methods for the analysis of high-throughput data in the context of the R programming environment [91]. Bioconductor is an ever-developing environment and at present it offers more than 3000 applications, including software for high-level statistical analysis, annotation, experimental data packages and specific workflows, designed for a variety of omics data.

In the era of precision medicine an increasing number of omics studies focus on identifying specific disease biomarkers from high-dimensional and heterogeneous datasets, and translating them to clinical practice. Concerning cardiovascular research, large quantities of biomedical, clinical and operational data are generated as part of patient care delivery, in addition to omics data coming from clinical and research laboratories. Therefore, to holistically confront complex diseases, it is crucial to take an approach that first combines multiomics with clinical data and/or experimental data (i.e., ‘data-integration’) and then extract hidden useful information [92,93].

Data-integration, which combines information across multiple data sources, promises to deliver more comprehensive insights into the biological system under study i.e., joining data from different domains such as genetics, genomics, transcriptomics, epigenomics, proteomics and metabolomics [94]. We report, for illustrative purposes, three novel methods for the integration of multiomics and clinical data: (i) the ‘mofa’ R package infers the best low-dimensional representation from multiomics datasets capturing the major source of variation across data and that can be correlated with clinical variables [95]. Alternatively, group samples can be identified by (ii) applying a partial least squares approach on several omics data layers in a supervised fashion, i.e., ‘MixOmics’, or (iii) build a patients’ similarity-based network, i.e., ‘NetDX’ [96,97].

Building robust and accurate prediction models for medicine needs machine learning approaches that allow the search for informative features within large data sets, possibly combining data from different omics layers [98]. To date, few tools have been specifically dedicated to the application of machine learning to omics data. Indeed, well-known statistical approaches for classification and regression, included in widely used software such as the ‘caret’ and ‘e1071′ R packages, were conceived to work with general, low dimensional datasets. To face this issue for transcriptomic data, two authors from this review have specifically developed open source tools, the R/Bioconductor packages DaMiRseq [99] and GARS [100], which perform comprehensive machine learning analysis starting from raw RNA-Seq data (adaptable also for microarray), e.g., normalizing data, identifying and removing known batch effects and latent source of variation, performing robust feature selection and building accurate classification models.

Following this great leap in technology development, the methods for the analysis of omics data and their integration require constant updating as well. This therefore calls for an interdisciplinary approach combining expertise from physicians, biologists and bioinformaticians.

## 7. Relevance of Omics for In Vitro DAC Modelling

Despite still being the main source of knowledge of dystrophinopathies, animal models are low throughput and often not representative of the human mechanisms, especially regarding cardiac involvement [13,101]. Considering the low availability of both skeletal and cardiac human tissue samples and the low proliferative activity of mature primary cardiomyocytes (CMs), iPSCs offer an interesting alternative. However, the main limit of applying iPSC-derived cardiomyocytes (iPSC-CMs) to model DAC is the inability to sufficiently guide cardiac differentiation and maturation. Moreover, compatibility with the high resolution of HTT, which are able to resolve individual disease phenotypes, calls for standardised and highly reproducible protocols (for more details the reader is directed to the following literature [102,103,104,105]). The path for a precision medicine approach to DAC depends on the ability of in vitro models to reproduce the patients’ clinical phenotype and to provide prognostic information.

In general, while the translation to more complex methods for research purposes is evolving (e.g., the decryption of specific molecular mechanisms in the context of MD skeletal and cardiac muscles) [106,107] it is far from reaching the ‘bed side’ (i.e., personalised medicine approach) or even towards the evaluation of novel pharmacological compounds with HTT.

The implementation of omics-based approaches could optimise the standardisation of advanced in vitro DAC models. First, omics could provide a quantitative description of the reproducibility of samples obtained from in vitro culture. iPSC-based culture, after the somatic cell reprogramming procedure, starts with the selection of clones considered representative of an ideal pluripotent state. However, making this selection free from arbitrariness, through a standardised multifactorial analysis, could be the first step towards the optimisation of the whole procedure [108,109,110]. Second, a deep understanding of the developmental trajectory leading to iPSC differentiation into specific cardiomyocyte subtypes or cardiovascular cell types, e.g., atrial cardiomyocytes or cardiac fibroblasts which both arise from second heart field progenitors [111,112], could guide standardised protocols and the interpretation of results. For instance, through a genomic approach, the relevance of activating specific mechanisms for in vitro maturation could be highlighted, as proposed by Friedman et al. [113], who demonstrated that hypertrophy and maturation of iPSC-CMs in vitro is secondary to the activation of the cardiac regulatory gene HOPX, suggestive of a possible quality check for cell cultures. Furthermore, the impact of the co-culture approach is supported by the work of Ruan et al. who used a scRNA-seq approach to underline a differentiation trajectory (involving Wnt-modulation), that demonstrated the supportive role of endothelial cells on CM maturation via ETS1 [114]. Similarly, tissue-specific fibroblasts and cardiac fibroblasts possess a reported capacity to enhance CM maturation in vitro [115,116]. Nevertheless, only a scRNA-seq approach enabled Zhang and colleagues [117] to untangle the regulatory pathways controlling differentiation of tissue-specific fibroblasts, thus paving the way to develop methods to obtain iPSC-derived cardiac fibroblasts. 

Omics approaches are also needed in order to describe the in vivo system, thus offering a control to demonstrate to what extent the in vitro model represents the in vivo scenario. For example, through a proteomics approach Doll and colleagues [118], obtained a map of the heart, describing the specificity of the cell types found in different cardiac regions and suggesting cell-surface markers representative of a pathological phenotype. In the same direction, but taking a step forward, Kamdar et al. [87] used transcriptome analysis to highlight that iPSC-CMs shared similarly dysregulated pathways with ex vivo primary cardiac cells. Thus, transcriptional profiling could follow in vitro experiments for drug testing, in order to identify the signalling pathways activated by potential beneficial drugs for DAC and possibly speeding their clinical translation [87,119].

Overall, exploitation of iPSC technology has the potential for disease modelling in general, and for DAC, in particular. However, it needs to take into account the complexity of living tissues, ranging from their multicellularity to their 3D structure [120]. This therefore calls for the design of advanced in vitro models that incorporate bioengineered solutions. Validation of these complex models necessitates the application of an integrated multiomic approach which to date remains missing.

## 8. Concluding Remarks

The effects of dystrophin deficiency on the skeletal muscles have been widely studied, thus therapeutic protocols and effective pharmacological treatments are available in clinical practice. More recently, the increase in the lifespan of patients with a dystrophinopathy encouraged focusing research on cardiac involvement. In particular, multiomic approaches promise substantial improvements towards understanding the underlying DAC mechanisms owing to increased information sourced from different disciplines that can promote our capacity to recapitulate the systemic complexity of DAC. 

Indeed, omics data generated through single techniques (transcriptomics, proteomics, metabolomics, etc.,) enable researchers to capture only a small part of a much more complex picture governing the molecular mechanisms that shape a particular phenotype. While the most commonly used approach is to analyse data from omics platforms independently from each other, modelling sets of different features through the use of multivariate approaches can leverage information obtained from diverse data types to provide a more accurate and in-depth picture of the system under study. With this integrative approach the existing relationships between different biomolecules, which can act in concert to modulate and influence biological systems and signalling pathways, could provide crucial biological information and increase the understanding of pathophysiological mechanisms. While being recently proposed for the study of the skeletal muscle involvement in MD [81], such an integrated approach is still missing for the study of DAC. Nevertheless, the findings summarised in Table 1 and Table 2 (proteomics and transcriptomics approach, respectively) suggest consistency between the biological mechanisms or pathways identified by the different studies (e.g., inflammation, ECM remodelling) that, although using different techniques, indicate the general feasibility of an integrated approach.

However, much work is still needed before this research proves clinically beneficial e.g., identifying relevant biomarkers for patient stratification, forming networked consortia with data-sharing agreements and common goals. One such effort has resulted in the creation of Muscle Gene Sets (http://www.sys-myo.com/muscle_gene_sets/; Accessed on 19 August 2021; also available via Enrichr, MsigDB/GSEA or WebGestalt), that provided a tool for functional genomics in neuromuscular conditions. While not yet extended to proteomic data, this tool offers the ability to study the behaviour of gene lists across more than 1100 comparisons of muscle conditions [121]. Extending the efforts of such a consortium by including tissue biobanks would provide the opportunity for using platform-type approaches for analysing multiomic data for DAC. As described in this review, omics, by generating and integrating multiomic quantitative data through advanced models, enables a multiscale and insightful overview of DAC pathology that offers enhanced possibilities capable of leading to a precision medicine approach. Meeting this objective will require leveraging the collective contributions of various stakeholders namely, representatives from MD patients’ groups, academia, industry and regulatory agencies [122].

## Figures and Tables

**Table 1:** Main studies performed on animal models of dystrophin deficiency using proteomic approach.

Model	Age	Methods	Findings	Reference
*mdx* mice	1,3,5,7,9 months	2D-DIGE and NMR	Altered mitochondrial and glycolytic enzymes	Gulston 2008
*mdx* mice	9 months	2D-DIGE	Altered energy metabolism, contraction and cytoskeletal proteins	Lewis 2010
*mdx* mice	7 weeks and 20 months	label free MS-based method	Reduced laminin, nidogen and annexin at 20 months	Holland 2013
*mdx*-4cv mice	20 months	label-free MS-based method	Altered basal lamina components, Ca^2+^-binding proteins, ECM proteins, cardiac myosin light chain kinase, stress response proteins and many mitochondrial and glycolytic enzymes	Murphy 2016
*mdx* mice	20 weeks	MS analysis of dystrophin interactors	Reduced association of dystrophin with nNOS and beta-1-syntrophin in the heart; altered cardiac ion flux and sarcomeric contraction	Johnson 2012
*mdx* mice	50 weeks	MS analysis of nitrosylated peptides	Increased mitochondrial and sarcomeric protein nitrosylation	Chung 2017
Pig	2 days and 3 months	Offgel prefractionation and iTRAQ-based MS analysis	Impaired mitochondrial energy production; increased inflammation; decreased DGC components	Tamiyakul 2020

2D-DIGE, two-dimensional difference gel electrophoresis; DGC, dystrophin-glycoprotein complex; ECM, extracellular matrix; iTRAQ, isobaric tag for relative and absolute quantitation; MS, mass spectrometry; NMR, nuclear magnetic resonance; nNOS, neuronal nitric oxide synthase.

**Table 2:** The main studies performed on dystrophin deficient models using transcriptomic approaches. DAC, Dystrophin-associated cardiomyopathy; DMD, Duchenne muscular dystrophy; ECM, extracellular matrix; iPSC, induced pluripotent stem cells; MS, mass spectrometry; RNA-seq, RNA-sequencing; snRNA-seq, single-nuclei RNA-sequencing; scRNA-seq, single-cell RNA-sequencing.

Model	Tissue	Method	Findings	Reference
human, DMD and alpha-sarcoglycan deficiency	muscle biopsy	microarray	developmental genes (e.g., alpha-actinin) overexpression; energy metabolism dysregulation; Ca^2+^ signalling; inflammation	Chen 2000
C57BL/10ScSn-*Dmd^mdx^*^/^J mouse, *mdx*	gastrocnemius and soleus muscle	microarray	upregulation of secreted phosphoprotein 1 (minopontin, osteopontin)	Porter 2002
human, DMD	skeletal muscle	microarray	overexpression of immune response signals and ECM genes	Haslett 2002
C57BL/10ScSn-*Dmd^mdx^*^/^J mouse, *mdx*	gastrocnemius and soleus muscle	microarray	ECM and inflammation transcript dysregulation	Porter 2003
C57BL/10ScSn-*Dmd^mdx^*^/^J B6Ros.Cg-*Dmd^mdx^*^−*5cv*^ mouse, *mdx*	diaphragm, extensor digitorum longus, gastrocnemius, quadriceps, soleus and tibialis anterior muscles	microarray	muscle specific molecular signatures explain fragility of muscle tissues	Haslett 2005
human, DMD	skeletal muscle	microarray	dystrophinopathy molecular signature characterised by genes involved in inflammation, ECM, muscle regeneration and energy metabolism	Pescatori 2007
C57BL/10ScSn-*Dmd^mdx^*^/^J and Fiona transgenic line mouse, *mdx*	Tibialis anterior muscle	microarray	utrophin modulates gene expression profile, thus is likely to be beneficial in dystrophin deficiency	Baban 2008
C57BL/10ScSn-*mdx* mouse, *mdx*	medial gastrocnemius	microarray	upregulated genes related to inflammation, ECM, muscle regeneration; identified candidate genes declining muscle necrosis, possible therapeutic targets;	Marotta 2009
human, DMD	skeletal muscle	RNA-seq	clinical genetic diagnostics, identification of intronic mutation	Gonorazky 2015
C57BL/10ScSn-*Dmd^mdx^*^/^J; B6C3Fe a/a-Large*^myd^*^/^J; and double mutant *Dmd^mdx^*^/^J/Large*^myd^*^/^J mouse, *mdx*	calf muscle	microarray	model signatures differ in genes regulating immune system, muscle degeneration/regeneration and ECM remodelling	Almeida 2016
C57/BL6N-*Dmd*ΔEx51 mouse	tibialis anterior muscle	RNA-seq; and snRNA-seq	myonuclei subtypes marker identification	Chemello 2020
human, DMD; and C57BL/10ScSn-*Dmd^mdx^*/J mouse, *mdx*	DMD patient-specific iPSC-derived cardiomyocytes; and mouse left ventricle	RNA-seq and scRNA-seq	assessment for DAC modelling, comparing profiling of DMD patients, with animal and in vitro models.	Kamdar 2020
mouse, *mdx/mTR^G2^*	plantaris and TA muscles	RNA-seq; Proteomics, metablomics lipidomics (MS)	DMD transcriptome and proteome signatures are different in protein balance, contractile elements, ECM and metabolism	Van Pelt 2021
human, DMD; and C57BL/10ScSn-*Dmd^mdx^*/J mouse, *mdx*	human and mouse blood	RNA-seq	DMD progression and therapy response evaluation in surrogate tissue	Signorelli 2021

## Data Availability

Not applicable.

## References

[1] Duan D., Goemans N., Takeda S., Mercuri E., Aartsma-Rus A. (2021). Duchenne muscular dystrophy. Nat. Rev. Dis. Primers.

[2] Constantin B. (2014). Dystrophin complex functions as a scaffold for signalling proteins. Biochim. Biophys. Acta.

[3] D’Amario D., Amodeo A., Adorisio R., Tiziano F.D., Leone A.M., Perri G., Bruno P., Massetti M., Ferlini A., Pane M. (2017). A current approach to heart failure in Duchenne muscular dystrophy. Heart.

[4] Flanigan K.M. (2012). The muscular dystrophies. Semin. Neurol..

[5] Monaco A.P., Bertelson C.J., Liechti-Gallati S., Moser H., Kunkel L.M. (1988). An explanation for the phenotypic differences between patients bearing partial deletions of the DMD locus. Genomics.

[6] Bushby K., Connor E. (2011). Clinical outcome measures for trials in Duchenne muscular dystrophy: Report from International Working Group meetings. Clin. Investig..

[7] Szabo S.M., Salhany R.M., Deighton A., Harwood M., Mah J., Gooch K.L. (2021). The clinical course of Duchenne muscular dystrophy in the corticosteroid treatment era: A systematic literature review. Orphanet J. Rare Dis..

[8] Finsterer J., Cripe L. (2014). Treatment of dystrophin cardiomyopathies. Nat. Rev. Cardiol..

[9] Verhaert D., Richards K., Rafael-Fortney J.A., Raman S.V. (2011). Cardiac involvement in patients with muscular dystrophies: Magnetic resonance imaging phenotype and genotypic considerations. Circ. Cardiovasc. Imaging.

[10] Melacini P., Vianello A., Villanova C., Fanin M., Miorin M., Angelini C., Dalla Volta S. (1996). Cardiac and respiratory involvement in advanced stage Duchenne muscular dystrophy. Neuromuscul. Disord..

[11] Tsuda T., Fitzgerald K.K. (2017). Dystrophic cardiomyopathy: Complex pathobiological processes to generate clinical phenotype. J. Cardiovasc. Dev. Dis..

[12] Vicente A.M., Ballensiefen W., Jonsson J.I. (2020). How personalised medicine will transform healthcare by 2030: The ICPerMed vision. J. Transl. Med..

[13] Gaina G., Popa Gruianu A. (2021). Muscular dystrophy: Experimental animal models and therapeutic approaches (Review). Exp. Ther. Med..

[14] McGreevy J.W., Hakim C.H., McIntosh M.A., Duan D. (2015). Animal models of Duchenne muscular dystrophy: From basic mechanisms to gene therapy. Dis. Model. Mech..

[15] Collins C.A., Morgan J.E. (2003). Duchenne’s muscular dystrophy: Animal models used to investigate pathogenesis and develop therapeutic strategies. Int. J. Exp. Pathol..

[16] Gonzalez A., Rota M., Nurzynska D., Misao Y., Tillmanns J., Ojaimi C., Padin-Iruegas M.E., Muller P., Esposito G., Bearzi C. (2008). Activation of cardiac progenitor cells reverses the failing heart senescent phenotype and prolongs lifespan. Circ. Res..

[17] Bulfield G., Siller W.G., Wight P.A., Moore K.J. (1984). X chromosome-linked muscular dystrophy (mdx) in the mouse. Proc. Natl. Acad. Sci. USA.

[18] Amoasii L., Long C., Li H., Mireault A.A., Shelton J.M., Sanchez-Ortiz E., McAnally J.R., Bhattacharyya S., Schmidt F., Grimm D. (2017). Single-cut genome editing restores dystrophin expression in a new mouse model of muscular dystrophy. Sci. Transl. Med..

[19] Young C.S., Mokhonova E., Quinonez M., Pyle A.D., Spencer M.J. (2017). Creation of a novel humanized dystrophic mouse model of duchenne muscular dystrophy and application of a CRISPR/Cas9 gene editing therapy. J. Neuromuscul. Dis..

[20] Min Y.L., Li H., Rodriguez-Caycedo C., Mireault A.A., Huang J., Shelton J.M., McAnally J.R., Amoasii L., Mammen P.P.A., Bassel-Duby R. (2019). CRISPR-Cas9 corrects Duchenne muscular dystrophy exon 44 deletion mutations in mice and human cells. Sci. Adv..

[21] Egorova T.V., Zotova E.D., Reshetov D.A., Polikarpova A.V., Vassilieva S.G., Vlodavets D.V., Gavrilov A.A., Ulianov S.V., Buchman V.L., Deykin A.V. (2019). CRISPR/Cas9-generated mouse model of Duchenne muscular dystrophy recapitulating a newly identified large 430 kb deletion in the human DMD gene. Dis. Model. Mech..

[22] Amoasii L., Li H., Zhang Y., Min Y.L., Sanchez-Ortiz E., Shelton J.M., Long C., Mireault A.A., Bhattacharyya S., McAnally J.R. (2019). In vivo non-invasive monitoring of dystrophin correction in a new Duchenne muscular dystrophy reporter mouse. Nat. Commun..

[23] Koo T., Lu-Nguyen N.B., Malerba A., Kim E., Kim D., Cappellari O., Cho H.Y., Dickson G., Popplewell L., Kim J.S. (2018). Functional rescue of dystrophin deficiency in mice caused by frameshift mutations using campylobacter jejuni Cas9. Mol. Ther..

[24] Ryu S.M., Koo T., Kim K., Lim K., Baek G., Kim S.T., Kim H.S., Kim D.E., Lee H., Chung E. (2018). Adenine base editing in mouse embryos and an adult mouse model of Duchenne muscular dystrophy. Nat. Biotechnol..

[25] Veltrop M., van Vliet L., Hulsker M., Claassens J., Brouwers C., Breukel C., van der Kaa J., Linssen M.M., den Dunnen J.T., Verbeek S. (2018). A dystrophic Duchenne mouse model for testing human antisense oligonucleotides. PLoS ONE.

[26] Nakamura K., Fujii W., Tsuboi M., Tanihata J., Teramoto N., Takeuchi S., Naito K., Yamanouchi K., Nishihara M. (2014). Generation of muscular dystrophy model rats with a CRISPR/Cas system. Sci. Rep..

[27] Larcher T., Lafoux A., Tesson L., Remy S., Thepenier V., Francois V., Le Guiner C., Goubin H., Dutilleul M., Guigand L. (2014). Characterization of dystrophin deficient rats: A new model for Duchenne muscular dystrophy. PLoS ONE.

[28] Miyamoto M., Tochinai R., Sekizawa S.I., Shiga T., Uchida K., Tsuru Y., Kuwahara M. (2020). Cardiac lesions in Duchenne muscular dystrophy model rats with out-of-frame Dmd gene mutation mediated by CRISPR/Cas9 system. J. Toxicol. Pathol..

[29] Sugihara H., Kimura K., Yamanouchi K., Teramoto N., Okano T., Daimon M., Morita H., Takenaka K., Shiga T., Tanihata J. (2020). Age-dependent echocardiographic and pathologic findings in a rat model with duchenne muscular dystrophy generated by CRISPR/Cas9 genome editing. Int. Heart J..

[30] Sui T., Lau Y.S., Liu D., Liu T., Xu L., Gao Y., Lai L., Li Z., Han R. (2018). A novel rabbit model of Duchenne muscular dystrophy generated by CRISPR/Cas9. Dis. Model. Mech..

[31] Chen Y., Zheng Y., Kang Y., Yang W., Niu Y., Guo X., Tu Z., Si C., Wang H., Xing R. (2015). Functional disruption of the dystrophin gene in rhesus monkey using CRISPR/Cas9. Hum. Mol. Genet..

[32] Wang S., Ren S., Bai R., Xiao P., Zhou Q., Zhou Y., Zhou Z., Niu Y., Ji W., Chen Y. (2018). No off-target mutations in functional genome regions of a CRISPR/Cas9-generated monkey model of muscular dystrophy. J. Biol. Chem..

[33] Aartsma-Rus A., Ginjaar I.B., Bushby K. (2016). The importance of genetic diagnosis for Duchenne muscular dystrophy. J. Med. Genet..

[34] Long C., Amoasii L., Mireault A.A., McAnally J.R., Li H., Sanchez-Ortiz E., Bhattacharyya S., Shelton J.M., Bassel-Duby R., Olson E.N. (2016). Postnatal genome editing partially restores dystrophin expression in a mouse model of muscular dystrophy. Science.

[35] Nelson C.E., Hakim C.H., Ousterout D.G., Thakore P.I., Moreb E.A., Castellanos Rivera R.M., Madhavan S., Pan X., Ran F.A., Yan W.X. (2016). In vivo genome editing improves muscle function in a mouse model of Duchenne muscular dystrophy. Science.

[36] Tabebordbar M., Zhu K., Cheng J.K.W., Chew W.L., Widrick J.J., Yan W.X., Maesner C., Wu E.Y., Xiao R., Ran F.A. (2016). In vivo gene editing in dystrophic mouse muscle and muscle stem cells. Science.

[37] Bengtsson N.E., Hall J.K., Odom G.L., Phelps M.P., Andrus C.R., Hawkins R.D., Hauschka S.D., Chamberlain J.R., Chamberlain J.S. (2017). Muscle-specific CRISPR/Cas9 dystrophin gene editing ameliorates pathophysiology in a mouse model for Duchenne muscular dystrophy. Nat. Commun..

[38] Xu L., Park K.H., Zhao L., Xu J., El Refaey M., Gao Y., Zhu H., Ma J., Han R. (2016). CRISPR-mediated Genome Editing Restores Dystrophin Expression and Function in mdx Mice. Mol. Ther..

[39] Long C., McAnally J.R., Shelton J.M., Mireault A.A., Bassel-Duby R., Olson E.N. (2014). Prevention of muscular dystrophy in mice by CRISPR/Cas9-mediated editing of germline DNA. Science.

[40] Zhang Y., Long C., Li H., McAnally J.R., Baskin K.K., Shelton J.M., Bassel-Duby R., Olson E.N. (2017). CRISPR-Cpf1 correction of muscular dystrophy mutations in human cardiomyocytes and mice. Sci. Adv..

[41] Zhu P., Wu F., Mosenson J., Zhang H., He T.C., Wu W.S. (2017). CRISPR/Cas9-mediated genome editing corrects dystrophin mutation in skeletal muscle stem cells in a mouse model of muscle dystrophy. Mol. Ther. Nucleic Acids.

[42] Lee K., Conboy M., Park H.M., Jiang F., Kim H.J., Dewitt M.A., Mackley V.A., Chang K., Rao A., Skinner C. (2017). Nanoparticle delivery of Cas9 ribonucleoprotein and donor DNA in vivo induces homology-directed DNA repair. Nat. Biomed. Eng..

[43] Amoasii L., Hildyard J.C.W., Li H., Sanchez-Ortiz E., Mireault A., Caballero D., Harron R., Stathopoulou T.R., Massey C., Shelton J.M. (2018). Gene editing restores dystrophin expression in a canine model of Duchenne muscular dystrophy. Science.

[44] Moretti A., Fonteyne L., Giesert F., Hoppmann P., Meier A.B., Bozoglu T., Baehr A., Schneider C.M., Sinnecker D., Klett K. (2020). Somatic gene editing ameliorates skeletal and cardiac muscle failure in pig and human models of Duchenne muscular dystrophy. Nat. Med..

[45] Colussi C., Banfi C., Brioschi M., Tremoli E., Straino S., Spallotta F., Mai A., Rotili D., Capogrossi M.C., Gaetano C. (2010). Proteomic profile of differentially expressed plasma proteins from dystrophic mice and following suberoylanilide hydroxamic acid treatment. Proteom. Clin. Appl..

[46] Gulston M.K., Rubtsov D.V., Atherton H.J., Clarke K., Davies K.E., Lilley K.S., Griffin J.L. (2008). A combined metabolomic and proteomic investigation of the effects of a failure to express dystrophin in the mouse heart. J. Proteome Res..

[47] Lewis C., Jockusch H., Ohlendieck K. (2010). Proteomic profiling of the dystrophin-deficient MDX heart reveals drastically altered levels of key metabolic and contractile proteins. J. Biomed. Biotechnol..

[48] Carr S.J., Zahedi R.P., Lochmuller H., Roos A. (2018). Mass spectrometry-based protein analysis to unravel the tissue pathophysiology in Duchenne muscular dystrophy. Proteom. Clin. Appl..

[49] Rouillon J., Zocevic A., Leger T., Garcia C., Camadro J.M., Udd B., Wong B., Servais L., Voit T., Svinartchouk F. (2014). Proteomics profiling of urine reveals specific titin fragments as biomarkers of Duchenne muscular dystrophy. Neuromuscul. Disord..

[50] Awano H., Matsumoto M., Nagai M., Shirakawa T., Maruyama N., Iijima K., Nabeshima Y.I., Matsuo M. (2018). Diagnostic and clinical significance of the titin fragment in urine of Duchenne muscular dystrophy patients. Clin. Chim. Acta.

[51] Robertson A.S., Majchrzak M.J., Smith C.M., Gagnon R.C., Devidze N., Banks G.B., Little S.C., Nabbie F., Bounous D.I., DiPiero J. (2017). Dramatic elevation in urinary amino terminal titin fragment excretion quantified by immunoassay in Duchenne muscular dystrophy patients and in dystrophin deficient rodents. Neuromuscul. Disord..

[52] Matsuo M., Awano H., Maruyama N., Nishio H. (2019). Titin fragment in urine: A noninvasive biomarker of muscle degradation. Adv. Clin. Chem..

[53] Rouillon J., Poupiot J., Zocevic A., Amor F., Leger T., Garcia C., Camadro J.M., Wong B., Pinilla R., Cosette J. (2015). Serum proteomic profiling reveals fragments of MYOM3 as potential biomarkers for monitoring the outcome of therapeutic interventions in muscular dystrophies. Hum. Mol. Genet..

[54] Brinkmeier H., Ohlendieck K. (2014). Chaperoning heat shock proteins: Proteomic analysis and relevance for normal and dystrophin-deficient muscle. Proteom. Clin. Appl..

[55] Holland A., Dowling P., Ohlendieck K. (2014). New pathobiochemical insights into dystrophinopathy from the proteomics of senescent mdx mouse muscle. Front. Aging Neurosci..

[56] Holland A., Dowling P., Zweyer M., Swandulla D., Henry M., Clynes M., Ohlendieck K. (2013). Proteomic profiling of cardiomyopathic tissue from the aged mdx model of Duchenne muscular dystrophy reveals a drastic decrease in laminin, nidogen and annexin. Proteomics.

[57] Holland A., Ohlendieck K. (2014). Proteomic profiling of the dystrophin-deficient mdx phenocopy of dystrophinopathy-associated cardiomyopathy. Biomed. Res. Int..

[58] Murphy S., Dowling P., Zweyer M., Mundegar R.R., Henry M., Meleady P., Swandulla D., Ohlendieck K. (2016). Proteomic analysis of dystrophin deficiency and associated changes in the aged mdx-4cv heart model of dystrophinopathy-related cardiomyopathy. J. Proteom..

[59] Johnson E.K., Zhang L., Adams M.E., Phillips A., Freitas M.A., Froehner S.C., Green-Church K.B., Montanaro F. (2012). Proteomic analysis reveals new cardiac-specific dystrophin-associated proteins. PLoS ONE.

[60] Li D., Yue Y., Lai Y., Hakim C.H., Duan D. (2011). Nitrosative stress elicited by nNOSmicro delocalization inhibits muscle force in dystrophin-null mice. J. Pathol..

[61] Chung H.S., Kim G.E., Holewinski R.J., Venkatraman V., Zhu G., Bedja D., Kass D.A., Van Eyk J.E. (2017). Transient receptor potential channel 6 regulates abnormal cardiac S-nitrosylation in Duchenne muscular dystrophy. Proc. Natl. Acad. Sci. USA.

[62] Murphy E., Kohr M., Sun J., Nguyen T., Steenbergen C. (2012). S-nitrosylation: A radical way to protect the heart. J. Mol. Cell. Cardiol..

[63] Zhang Y., Deng Y., Yang X., Xue H., Lang Y. (2020). The relationship between protein S-nitrosylation and human diseases: A review. Neurochem. Res..

[64] Tamiyakul H., Kemter E., Kosters M., Ebner S., Blutke A., Klymiuk N., Flenkenthaler F., Wolf E., Arnold G.J., Frohlich T. (2020). Progressive proteome changes in the myocardium of a pig model for duchenne muscular dystrophy. iScience.

[65] Judge D.P., Kass D.A., Thompson W.R., Wagner K.R. (2011). Pathophysiology and therapy of cardiac dysfunction in Duchenne muscular dystrophy. Am. J. Cardiovasc. Drugs.

[66] Diegoli M., Grasso M., Favalli V., Serio A., Gambarin F.I., Klersy C., Pasotti M., Agozzino E., Scelsi L., Ferlini A. (2011). Diagnostic work-up and risk stratification in X-linked dilated cardiomyopathies caused by dystrophin defects. J. Am. Coll. Cardiol..

[67] Shirokova N., Niggli E. (2013). Cardiac phenotype of Duchenne Muscular Dystrophy: Insights from cellular studies. J. Mol. Cell. Cardiol..

[68] Caspi T., Straw S., Cheng C., Garnham J.O., Scragg J.L., Smith J., Koshy A.O., Levelt E., Sukumar P., Gierula J. (2020). Unique transcriptome signature distinguishes patients with heart failure with myopathy. J. Am. Heart Assoc..

[69] Chiesa M., Piacentini L., Bono E., Milazzo V., Campodonico J., Marenzi G., Colombo G.I. (2020). Whole blood transcriptome profile at hospital admission discriminates between patients with ST-segment elevation and non-ST-segment elevation acute myocardial infarction. Sci. Rep..

[70] Piacentini L., Werba J.P., Bono E., Saccu C., Tremoli E., Spirito R., Colombo G.I. (2019). Genome-wide expression profiling unveils autoimmune response signatures in the perivascular adipose tissue of abdominal aortic aneurysm. Arterioscler. Thromb. Vasc. Biol..

[71] Chen Y.W., Zhao P., Borup R., Hoffman E.P. (2000). Expression profiling in the muscular dystrophies: Identification of novel aspects of molecular pathophysiology. J. Cell Biol..

[72] Haslett J.N., Sanoudou D., Kho A.T., Bennett R.R., Greenberg S.A., Kohane I.S., Beggs A.H., Kunkel L.M. (2002). Gene expression comparison of biopsies from Duchenne muscular dystrophy (DMD) and normal skeletal muscle. Proc. Natl. Acad. Sci. USA.

[73] Pescatori M., Broccolini A., Minetti C., Bertini E., Bruno C., D’Amico A., Bernardini C., Mirabella M., Silvestri G., Giglio V. (2007). Gene expression profiling in the early phases of DMD: A constant molecular signature characterizes DMD muscle from early postnatal life throughout disease progression. FASEB J..

[74] Marotta M., Ruiz-Roig C., Sarria Y., Peiro J.L., Nunez F., Ceron J., Munell F., Roig-Quilis M. (2009). Muscle genome-wide expression profiling during disease evolution in mdx mice. Physiol. Genom..

[75] Porter J.D., Khanna S., Kaminski H.J., Rao J.S., Merriam A.P., Richmonds C.R., Leahy P., Li J., Guo W., Andrade F.H. (2002). A chronic inflammatory response dominates the skeletal muscle molecular signature in dystrophin-deficient mdx mice. Hum. Mol. Genet..

[76] Porter J.D., Merriam A.P., Leahy P., Gong B., Khanna S. (2003). Dissection of temporal gene expression signatures of affected and spared muscle groups in dystrophin-deficient (mdx) mice. Hum. Mol. Genet..

[77] Baban D., Davies K.E. (2008). Microarray analysis of mdx mice expressing high levels of utrophin: Therapeutic implications for dystrophin deficiency. Neuromuscul. Disord..

[78] Almeida C.F., Martins P.C., Vainzof M. (2016). Comparative transcriptome analysis of muscular dystrophy models Large(myd), Dmd(mdx)/Large(myd) and Dmd(mdx): What makes them different?. Eur. J. Hum. Genet..

[79] Haslett J.N., Kang P.B., Han M., Kho A.T., Sanoudou D., Volinski J.M., Beggs A.H., Kohane I.S., Kunkel L.M. (2005). The influence of muscle type and dystrophin deficiency on murine expression profiles. Mamm. Genome.

[80] Byron S.A., Van Keuren-Jensen K.R., Engelthaler D.M., Carpten J.D., Craig D.W. (2016). Translating RNA sequencing into clinical diagnostics: Opportunities and challenges. Nat. Rev. Genet..

[81] Van Pelt D.W., Kharaz Y.A., Sarver D.C., Eckhardt L.R., Dzierzawski J.T., Disser N.P., Piacentini A.N., Comerford E., McDonagh B., Mendias C.L. (2021). Multiomics analysis of the mdx/mTR mouse model of Duchenne muscular dystrophy. Connect. Tissue Res..

[82] Paik D.T., Cho S., Tian L., Chang H.Y., Wu J.C. (2020). Single-cell RNA sequencing in cardiovascular development, disease and medicine. Nat. Rev. Cardiol..

[83] Stahl P.L., Salmen F., Vickovic S., Lundmark A., Navarro J.F., Magnusson J., Giacomello S., Asp M., Westholm J.O., Huss M. (2016). Visualization and analysis of gene expression in tissue sections by spatial transcriptomics. Science.

[84] Liao J., Lu X., Shao X., Zhu L., Fan X. (2021). Uncovering an organ’s molecular architecture at single-cell resolution by spatially resolved transcriptomics. Trends Biotechnol..

[85] Chemello F., Wang Z., Li H., McAnally J.R., Liu N., Bassel-Duby R., Olson E.N. (2020). Degenerative and regenerative pathways underlying Duchenne muscular dystrophy revealed by single-nucleus RNA sequencing. Proc. Natl. Acad. Sci. USA.

[86] Gonorazky H., Liang M., Cummings B., Lek M., Micallef J., Hawkins C., Basran R., Cohn R., Wilson M.D., MacArthur D. (2016). RNAseq analysis for the diagnosis of muscular dystrophy. Ann. Clin. Transl. Neurol..

[87] Kamdar F., Das S., Gong W., Klaassen Kamdar A., Meyers T.A., Shah P., Ervasti J.M., Townsend D., Kamp T.J., Wu J.C. (2020). Stem cell-derived cardiomyocytes and beta-adrenergic receptor blockade in duchenne muscular dystrophy cardiomyopathy. J. Am. Coll. Cardiol..

[88] Signorelli M., Ebrahimpoor M., Veth O., Hettne K., Verwey N., Garcia-Rodriguez R., Tanganyika-deWinter C.L., Lopez Hernandez L.B., Escobar Cedillo R., Gomez Diaz B. (2021). Peripheral blood transcriptome profiling enables monitoring disease progression in dystrophic mice and patients. EMBO Mol. Med..

[89] Rosenberg A.S., Puig M., Nagaraju K., Hoffman E.P., Villalta S.A., Rao V.A., Wakefield L.M., Woodcock J. (2015). Immune-mediated pathology in Duchenne muscular dystrophy. Sci. Transl. Med..

[90] Mendell J.R., Campbell K., Rodino-Klapac L., Sahenk Z., Shilling C., Lewis S., Bowles D., Gray S., Li C., Galloway G. (2010). Dystrophin immunity in Duchenne’s muscular dystrophy. N. Engl. J. Med..

[91] Gentleman R.C., Carey V.J., Bates D.M., Bolstad B., Dettling M., Dudoit S., Ellis B., Gautier L., Ge Y., Gentry J. (2004). Bioconductor: Open software development for computational biology and bioinformatics. Genome Biol..

[92] Subramanian I., Verma S., Kumar S., Jere A., Anamika K. (2020). Multi-omics data integration, interpretation, and its application. Bioinform. Biol. Insights.

[93] Shameer K., Johnson K.W., Glicksberg B.S., Dudley J.T., Sengupta P.P. (2018). Machine learning in cardiovascular medicine: Are we there yet?. Heart.

[94] Hasin Y., Seldin M., Lusis A. (2017). Multi-omics approaches to disease. Genome Biol..

[95] Argelaguet R., Velten B., Arnol D., Dietrich S., Zenz T., Marioni J.C., Buettner F., Huber W., Stegle O. (2018). Multi-Omics factor analysis-a framework for unsupervised integration of multi-omics data sets. Mol. Syst. Biol..

[96] Rohart F., Gautier B., Singh A., Le Cao K.A. (2017). mixOmics: An R package for ‘omics feature selection and multiple data integration. PLoS Comput. Biol..

[97] Pai S., Hui S., Isserlin R., Shah M.A., Kaka H., Bader G.D. (2019). netDx: Interpretable patient classification using integrated patient similarity networks. Mol. Syst. Biol..

[98] Mirza B., Wang W., Wang J., Choi H., Chung N.C., Ping P. (2019). Machine learning and integrative analysis of biomedical big data. Genes.

[99] Chiesa M., Colombo G.I., Piacentini L. (2018). DaMiRseq-an R/Bioconductor package for data mining of RNA-Seq data: Normalization, feature selection and classification. Bioinformatics.

[100] Chiesa M., Maioli G., Colombo G.I., Piacentini L. (2020). GARS: Genetic Algorithm for the identification of a robust subset of features in high-dimensional datasets. BMC Bioinform..

[101] Heydemann A., Wheeler M.T., McNally E.M. (2001). Cardiomyopathy in animal models of muscular dystrophy. Curr. Opin. Cardiol..

[102] Karbassi E., Fenix A., Marchiano S., Muraoka N., Nakamura K., Yang X., Murry C.E. (2020). Cardiomyocyte maturation: Advances in knowledge and implications for regenerative medicine. Nat. Rev. Cardiol..

[103] Ahmed R.E., Anzai T., Chanthra N., Uosaki H. (2020). A brief review of current maturation methods for human induced pluripotent stem cells-derived cardiomyocytes. Front. Cell Dev. Biol..

[104] Guo Y., Pu W.T. (2020). Cardiomyocyte maturation: New phase in development. Circ. Res..

[105] Maroli G., Braun T. (2021). The long and winding road of cardiomyocyte maturation. Cardiovasc. Res..

[106] Smith A.S.T., Davis J., Lee G., Mack D.L., Kim D.H. (2016). Muscular dystrophy in a dish: Engineered human skeletal muscle mimetics for disease modeling and drug discovery. Drug Discov. Today.

[107] Caputo L., Granados A., Lenzi J., Rosa A., Ait-Si-Ali S., Puri P.L., Albini S. (2020). Acute conversion of patient-derived Duchenne muscular dystrophy iPSC into myotubes reveals constitutive and inducible over-activation of TGFbeta-dependent pro-fibrotic signaling. Skelet. Muscle.

[108] Volpato V., Webber C. (2020). Addressing variability in iPSC-derived models of human disease: Guidelines to promote reproducibility. Dis. Model Mech..

[109] Musunuru K., Sheikh F., Gupta R.M., Houser S.R., Maher K.O., Milan D.J., Terzic A., Wu J.C., Translational B., American Heart Association Council on Functional, G. (2018). Induced pluripotent stem cells for cardiovascular disease modeling and precision medicine: A scientific statement from the american heart association. Circ. Genom. Precis. Med..

[110] Biendarra-Tiegs S.M., Secreto F.J., Nelson T.J. (2020). Addressing variability and heterogeneity of induced pluripotent stem cell-derived cardiomyocytes. Adv. Exp. Med. Biol..

[111] Mesquita F.C.P., Morrissey J., Lee P.F., Monnerat G., Xi Y., Andersson H., Nogueira F.C.S., Domont G.B., Sampaio L.C., Hochman-Mendez C. (2021). Cues from human atrial extracellular matrix enrich the atrial differentiation of human induced pluripotent stem cell-derived cardiomyocytes. Biomater. Sci..

[112] Zhang J., Tao R., Campbell K.F., Carvalho J.L., Ruiz E.C., Kim G.C., Schmuck E.G., Raval A.N., da Rocha A.M., Herron T.J. (2019). Functional cardiac fibroblasts derived from human pluripotent stem cells via second heart field progenitors. Nat. Commun..

[113] Friedman C.E., Nguyen Q., Lukowski S.W., Helfer A., Chiu H.S., Miklas J., Levy S., Suo S., Han J.J., Osteil P. (2018). Single-cell transcriptomic analysis of cardiac differentiation from human PSCs reveals HOPX-dependent cardiomyocyte maturation. Cell Stem Cell.

[114] Ruan H., Liao Y., Ren Z., Mao L., Yao F., Yu P., Ye Y., Zhang Z., Li S., Xu H. (2019). Single-cell reconstruction of differentiation trajectory reveals a critical role of ETS1 in human cardiac lineage commitment. BMC Biol..

[115] Giacomelli E., Meraviglia V., Campostrini G., Cochrane A., Cao X., van Helden R.W.J., Krotenberg Garcia A., Mircea M., Kostidis S., Davis R.P. (2020). Human-iPSC-derived cardiac stromal cells enhance maturation in 3D cardiac microtissues and reveal non-cardiomyocyte contributions to heart disease. Cell Stem Cell.

[116] Beauchamp P., Jackson C.B., Ozhathil L.C., Agarkova I., Galindo C.L., Sawyer D.B., Suter T.M., Zuppinger C. (2020). 3D Co-culture of hiPSC-derived cardiomyocytes with cardiac fibroblasts improves tissue-like features of cardiac spheroids. Front. Mol. Biosci..

[117] Zhang H., Tian L., Shen M., Tu C., Wu H., Gu M., Paik D.T., Wu J.C. (2019). Generation of quiescent cardiac fibroblasts from human induced pluripotent stem cells for in vitro modeling of cardiac fibrosis. Circ. Res..

[118] Doll S., Dressen M., Geyer P.E., Itzhak D.N., Braun C., Doppler S.A., Meier F., Deutsch M.A., Lahm H., Lange R. (2017). Region and cell-type resolved quantitative proteomic map of the human heart. Nat. Commun..

[119] Sun C., Choi I.Y., Rovira Gonzalez Y.I., Andersen P., Talbot C.C., Iyer S.R., Lovering R.M., Wagner K.R., Lee G. (2020). Duchenne muscular dystrophy hiPSC-derived myoblast drug screen identifies compounds that ameliorate disease in mdx mice. JCI Insight.

[120] Maffioletti S.M., Sarcar S., Henderson A.B.H., Mannhardt I., Pinton L., Moyle L.A., Steele-Stallard H., Cappellari O., Wells K.E., Ferrari G. (2018). Three-dimensional human iPSC-derived artificial skeletal muscles model muscular dystrophies and enable multilineage tissue engineering. Cell Rep..

[121] Malatras A., Duguez S., Duddy W. (2019). Muscle gene sets: A versatile methodological aid to functional genomics in the neuromuscular field. Skelet. Muscle.

[122] Straub V., Balabanov P., Bushby K., Ensini M., Goemans N., De Luca A., Pereda A., Hemmings R., Campion G., Kaye E. (2016). Stakeholder cooperation to overcome challenges in orphan medicine development: The example of Duchenne muscular dystrophy. Lancet Neurol..

